# Mental Health Status of High School Students in Khartoum State, Sudan During the COVID-19 Pandemic: A Cross-Sectional Study

**DOI:** 10.1192/bjo.2022.164

**Published:** 2022-06-20

**Authors:** Randa Altamih, Osman Elmahi

**Affiliations:** 1 Faculty of Medicine, University of Khartoum, Khartoum, Sudan; 2 Faculty of Medicine, Ibn Sina University, Khartoum, Sudan

## Abstract

**Aims:**

This study sought to assess mental health status of high school students in Khartoum State, to evaluate the participants’ adherence to COVID-19 preventive measures and to identify factors associated with commitment to COVID-19 guidelines and mental health status during the COVID-19 pandemic.

**Methods:**

This was a descriptive, cross-sectional and institution-based study. 364 post-primary students in 10 schools were selected by multistage stratified cluster sampling. Mental health status was evaluated using the General Health Questionnaire (GHQ-12). Chi-square testing was used to identify influencing factors of mental health status and commitment to practicing COVID-19 preventive measures.

**Results:**

A median commitment score of 2/5 was achieved. 70.8% of students in this study demonstrated low commitment (< 50%) to practicing COVID-19 preventive guidelines. Commitment to COVID-19 preventive measures was significantly associated with gender (p = 0.047), academic year (p = 0.033) and post-primary schools attended by the participants (p < 0.001). 36.5% of the participants’ GHQ-12 scores demonstrated severe psychological distress (GHQ-12 > 20/36). A mean GHQ-12 score of 18.4 and median of 19 was achieved, indicating moderate to severe psychological distress. GHQ-12 scores were significantly associated with incidence of COVID-19 infection among family members (p = 0.016).

**Conclusion:**

Over one-third of high school students in Khartoum State demonstrated moderate to severe psychological distress as a result of the COVID-19 pandemic, which was significantly associated with first-degree family members having a confirmed COVID-19 diagnosis. Commitment to preventive measures set by the General Directorate of Global Health was significantly associated with gender and academic year. A lesser psychological impact could be achieved through timely health education, expression of confidence in professional healthcare providers and perception of sound health status, together with consistent public health campaigning.

